# Predictors for mortality in hospitalized patients with chronic obstructive pulmonary disease

**DOI:** 10.1186/2193-1801-3-359

**Published:** 2014-07-15

**Authors:** Yan Cheng, Matthew E Borrego, Floyd J Frost, Hans Petersen, Dennis W Raisch

**Affiliations:** Department of Pharmacotherapy, University of Utah, Salt Lake City, Utah USA; Pharmacoeconomics, Epidemiology, Public Policy and Outcome Research, College of Pharmacy, University of New Mexico, Albuquerque, NM USA; Department of Family and Community Medicine, School of Medicine, University of New Mexico, Albuquerque, NM USA; Lovelace Respiratory Research Institute, Albuquerque, NM USA

**Keywords:** Chronic obstructive pulmonary disease, Mortality, Predictor

## Abstract

**Background:**

Chronic obstructive pulmonary disease (COPD) has been the only leading cause of death associated with a continuously increasing trend in the US over the past 30 years.

**Objectives:**

The aim of this research was to identify predictors for all-cause in-hospital mortality for COPD patients.

**Methods:**

We conducted a cross-sectional study of patients with the discharge diagnosis of COPD, utilizing the 2007 Premier Perspective database. Inpatients aged 40 years and above were selected if they had a discharge with a primary diagnosis of COPD between January 1, 2007 and December 31, 2007. All data analyses were based on individual level. If a patient had multiple discharges, only the last discharge was included for mortality analysis. Predictors for mortality were identified by multiple logistic regressions. Bonferroni correction for multiple logistic regression models was adapted to control for family-wise errors.

**Results:**

The total of 57,224 patients was selected for data analysis in the study. All-cause in-hospital mortality for patients with COPD was 2.4%. Older age, insurance coverage, elective admission, intensive care unit admission, prolonged length of stay, increased Deyo-adapted Charlson Index (DCI) score and Elixhauser comorbidities of renal failure, metastatic cancer, solid tumor without metastasis, and weight loss were identified as independent predictors for all-cause in-hospital mortality. Antibiotics and β-blockers were predictors of lower all-cause in-hospital mortality risk after adjusting for other factors.

**Conclusions:**

The nationwide discharge database provides useful information to identify predictors for all-cause in-hospital mortality of patients with COPD.

**Electronic supplementary material:**

The online version of this article (doi:10.1186/2193-1801-3-359) contains supplementary material, which is available to authorized users.

## Background

Chronic obstructive pulmonary disease (COPD) has been ranked as the 3rd leading cause of death in the US only exceeded by heart disease and cancer since 2008 (Minino and Kochanek 2010). A recently released report on the trends in COPD morbidity and mortality of the US populations indicated that the number of death caused by COPD increased from 119,524 in 1999 to 133,965 in 2009 (American Lung Association 2013). It is estimated that COPD has been the only leading cause of death associated with a continuously increasing trend in the US over the past 30 years (COPDGene 2010).

Since COPD is a preventable and treatable disease, it is important to identify the predictors associated with higher mortality in patients with COPD so that we can take measures to reduce their risk of death. A review by Steer et al. discovered that older age, lower diminished forced expiratory volume in one second (FEV1), current smoking, prior hospitalization, poor nutrition status, as well as specific chronic comobidities were the predictors for both in-hospital and post-discharge mortality; and acute comorbidites were only independently associated with in-hospital mortality (Steer et al. 2010). Another recently published systematic review reported that some specific comorbid conditions, such as low body mass index (BMI) and cardiac failure, were significantly associated with both long-term and short-term in-hospital mortality (Singanayagam et al. 2013). However, there has been a controversy whether a routinely used comorbidity measure such as the Charlson Comorbidity Index can help predict mortality in COPD patients. Previous studies have reported the Charlson Comorbidity Index to be a significant predictor of mortality in COPD patients (Almagro et al. 2002; Casanova et al. 2005; Casanova et al. 2008; De Torres et al. 2009; Fan et al. 2002; Marti et al. 2006; Patil et al. 2003), while others did not find this association (Bustamante-Fermosel et al. 2007; Celli et al. 2004; Kim et al. 2006; Pinto-Plata et al. 2004; Ranieri et al. 2008; Soler-Cataluna 2005; Soler-Cataluna et al. 2009; Terzano et al. 2010). Conversely, the Exlihauser Comorbidity measure, another comorbidity algorithm, is rarely used to predict mortality risk in patients with COPD.

In this study, we explored the factors associated with all-cause, in-hospital mortality during COPD related hospital admissions based on a nationally representative, administrative hospital discharge database. We assessed: (1) associations between mortality and patient socio-demographic characteristics (i.e., age, race, gender, insurance plan) and hospital-related characteristics (i.e., hospital location, hospital type, number of beds, admission type, length of stay (LOS)); (2) association between mortality and patient's medical characteristics (i.e. Elixhauser comorbidity measure, Deyo-adapted Charlson Comorbidity Index (DCI), All Patient Refined Diagnosis Related Groups (APR-DRG^a^) severity of illness, and APR-DRG risk of mortality); and (3) relationships between mortality and the use of drug therapies (β-blockers, oral/parenteral corticosteroids, and/or antibiotics) during a hospital stay, after adjusting for other variables.

## Methods

We utilized the 2007 Premier Perspective database to conduct a retrospective, cross-sectional study in patients with the ICD-9-CM discharge diagnoses of COPD. The Premier Perspective database and its participants represent a nationwide group of hospitals which are willing and have infrastructure to submit and track the data needed for decisions about Medicare and Medicaid payments (Premier 2010). The Premier database used for the study included patient demographics, insurance status, hospital characteristics, primary and secondary discharge diagnoses, and pharmacotherapy. Inpatient discharges with a primary COPD ICD-9 diagnosis code (491.XX, chronic bronchitis; 492.X, emphysema; or 496, chronic airway obstruction not elsewhere classified) between January 1, 2007 and December 31, 2007 were selected for this study. We excluded patients younger than 40 years for the consideration of misclassification of disease status (American Thoracic Society 1995). Inpatient discharges missing required any information for the variables used in the study were also excluded. The analyses were conducted at the patient level. If a patient had multiple discharges in the database, only the last discharge was included.

The outcome was all-cause, in-hospital mortality in COPD patients defined as a dichotomous variable with values 0 (survived) or 1 (died). Independent variables included age, gender, race, insurance status, hospital location, hospital type, number of beds, admission type, ICU admission, length of stay (LOS), comorbidities (Elixhauser comorbidities or DCI), APR-DRG severity of illness, APR-DRG risk of mortality, and pharmacotherapy. The pharmacotherapy variable included the use of β-blockers, oral/parenteral corticosteroids, or antibiotics during the hospital stay.

Age was treated as a continuous variable. Race was categorized as Caucasian, African American, Hispanic, Asian, American Indian, or other. Insurance status included Medicare, Medicaid, managed care, commercial, other (workers compensation, direct employer contract, and other government payers), and uninsured. Hospital location was defined as rural and urban. Hospital type was defined as teaching and non-teaching. Number of beds was defined as total number of beds in hospital. Admission type included emergency, urgent, elective and trauma. ICU admission was a dichotomous variable, ICU admission and non-ICU admission. LOS was a count, calculated in days. Comorbid conditions were identified by the Elixhauser Comorbidity Software, Version 3.1 using both DRG and ICD-9-CM for each patient per discharge. The DCI index was based on 17 diagnostic categories identified from ICD-9-CM discharge diagnoses. Each comorbid condition was assigned a weight from the range of (1,2,3,6). The DCI index was categorized into five groups: 0, 1, 2, 3, 4+. APR-DRG severity of illness was defined as severity level of each specific DRG, reflecting the extent of physiologic decompensation or organ system loss of function and classified into four degrees (minor, moderate, major, extreme) (Hughes and Development of the 3M™ All Patient Refined Diagnosis Related Groups APR DRGs 2012) APR-DRG risk of mortality was defined as the likelihood of dying from a specific DRG (minor, moderate, major and extreme) (Hughes and Development of the 3M™ All Patient Refined Diagnosis Related Groups APR DRGs 2012). Pharmacotherapy included only β-blockers, oral/parenteral corticosteroids, and antibiotics because published literature indicates these are important treatments for COPD (Rutten et al. 2010; Schols et al. 2001; Fan et al. 2003; Lee et al. 2009; Lee et al. 2008; Dransfield et al. 2008; Rothberg et al. 2010). Each therapy was created as a dichotomous variable with values 0 (not used) and 1 (used).

Collinearity tests and Spearman correlation coefficients were used to assess the multilinearity between the independent variables. For each specific DRG, the outliers of LOS were identified if an observed value for LOS was larger than the population mean plus 4 standard deviations according to the “68-95-99.7 rule” (68-95-99.7 rule 2012).

We conducted chi-square analyses and t-tests to identify the association between mortality and each independent variable, and calculated the odds ratio for each independent variable by simple logistic regression. A stepwise multiple logistic regression was conducted to identify significant independent variables predicting all cause, in-hospital mortality in COPD patients after adjusting for other covariates. All statistical analyses were performed using the SAS statistical program, version 9.2 for Windows (SAS Institute, Cary, NC). For all statistical tests the two sided alpha level was 0.05. Bonferroni correction was adapted to control the familywise errors for multiple logistic regression models. Assuming 20 predictors for outcome death, p values < 0.0025 were considered significant in this analysis.

## Results

### Characteristics of the studied populations

The 2007 Premier database contained 71,808 discharges associated with the International Classification of Disease, Ninth Revision, Clinical Modification (ICD-9-CM) primary diagnosis of COPD. After we applied inclusion and exclusion criteria, 69,464 discharges remained, which represented 57,224 unique individuals. A total of 895 patients had the last two discharges in the same month, and 65 of the 895 died in hospital. The remainder of 830 patients was excluded because it was not possible to determine which admission was the last in the month. After excluding those individuals, there were 56,394 unique patients available for analysis, represented by 86.9% individuals who had one discharge, 9.5% had two, 2.3% had three, and 1.3% had 4 to 13 discharges in 2007. A total of 1,361 patients died during hospitalization, thus the all cause, in-hospital mortality rate in COPD patients was 2.4%. The mean age was 69.7 (SD 11.7) years, 43.4% were males, and 73.8% were Caucasian. The median LOS was 4 (interquartile range [IQR] 2–6) days.

Among the 56,394 unique patients, 99.83% (n = 56,390) had non-zero DRG codes and 0.17% (n = 94) had code 0. For each non-zero DRG code, we calculated the mean and standard deviation of LOS (Additional file 1). The observations which had a value for LOS larger than the mean for that DRG plus 4 times standard deviation were regarded as outliers and were excluded from the dataset, because these patients are unique according to Medicare DRG criteria and could bias the results. The observations with DRG code 0 were also excluded as the outliers, since there should have not been the value “0” for DRG code. Therefore, 427 patients were excluded resulting in 55,967 patients remaining for the mortality analysis.

The chi-square analyses and t-tests are shown in Table 1. Patients who died were older than those who survived with the mean age of 69.6 v.s. 74.4 years. The both groups had a higher percentage of females than males, but the proportion of males in patients who died were even higher. Compared to patients who survived, patients who died had a higher proportion of Caucasians and Asians but a lower proportion of African Americans, Hispanics, and American Indians. The LOS was longer in patients who died than patients who survived with the mean value of 4.7 v.s. 9.0 days. Patients who died had a much higher proportion of ICU admissions and a higher DCI than those who survived.

**Table 1:** **Description of the studied samples (N = 56,394)**

Characteristics	Patients (survived)	Patients (died)	P - value
Age (mean ± s.d.)	69.6 ± 11.7	74.4 ± 9.5	<0.001
Gender			
Male	23,842 (43.3%)	658 (48.4%)	<0.001
Female	31,191 (56.7%)	703 (51.5%)	
Race/ethnicity			
Caucasian	40,554 (73.7%)	1,035 (76.1%)	<0.001
African American	5,356 (9.7%)	104 (7.6%)	
Hispanic	1,331 (2.4%)	14 (1.0%)	
American Indian	304 (0.6%)	2 (0.2%)	
Asian	451 (0.8%)	19 (1.4%)	
Other race	7,037 (12.8%)	187 (13.7%)	
Insurance type			
Medicare	40,130 (72.9%)	1,045 (76.8%)	<0.001
Medicaid	4,225 (7.7%)	49 (3.6%)	
Managed care	5,742 (10.4%)	82 (6.0%)	
Commercial	2,050 (3.7%)	77 (5.7%)	
Other	1,056 (1.9%)	86 (6.3%)	
Uninsured	1,830 (3.3%)	22 (1.6%)	
Hospitals			
Urban	46,212 (84.0%)	1,157 (85.0%)	0.301
Rural	8,821 (16.0%)	204 (15.0%)	
Teaching	18,722 (34.0%)	467 (34.3%)	0.821
Non-teaching	36,311 (66.0%)	894 (65.7%)	
Number of beds (mean ± s.d.)	398.1 ± 222.8	393.7 ± 215.9	0.465
LOS (mean ± s.d.)	4.7 ± 4.1	9.0 ± 9.2	<0.001
Number of discharges (mean ± s.d.)	1.2 ± 0.6	1.3 ± 0.8	<0.001
Admission type			
Emergency	40,555 (73.7%)	906 (66.6%)	<0.001
Urgent	9,307 (17.0%)	236 (17.3%)	
Elective	4,954 (9.0%)	213 (15.7%)	
Trauma	217 (0.4%)	6 (0.4%)	
ICU admission	4,521 (8.2%)	600 (44.1%)	<0.001
APR-DRG severity of illness			
Minor	10,616 (19.3%)	67 (4.9%)	<0.001
Moderate	24,659 (44.8%)	218 (16.0%)	
Major	17,176 (31.2%)	438 (32.2%)	
Extreme	2,582 (4.7%)	638 (46.9%)	
APR-DRG risk of mortality			
Minor	20,979 (38.1%)	64 (4.7%)	<0.001
Moderate	21,900 (39.8%)	301 (22.1%)	
Major	10,249 (18.6%)	476 (35.0%)	
Extreme	1,905 (3.5%)	520 (38.2%)	
Drug therapies			
Oral/parenteral corticosteroids	46,990 (85.4%)	1,116 (82.0%)	<0.001
Antibiotics	48,132 (87.5%)	1,122 (82.4%)	<0.001
β-blockers	18,143 (33.0%)	448 (32.9%)	0.969
DCI score			
0	22,614 (41.1%)	360 (26.5%)	<0.001
1	15,013 (27.3%)	373 (27.4%)	
2	8,558 (15.6%)	250 (18.4%)	
3	4,319 (7.9%)	156 (11.5%)	
4+	4,529 (8.2%)	222 (16.3%)	
Elixhauser comorbidity*			
Valvular disease	1,027 (1.9%)	36 (2.7%)	0.037
Paralysis	119 (0.22%)	2 (0.2%)	0.803**
Neurological disorders	1,175 (2.1%)	32 (2.4%)	0.586
Other chronic lung disease	97 (0.2%)	17 (1.3%)	<0.001
Hypothyroidism	6,969 (12.7%)	143 (10.5%)	0.018
Renal failure	54 (0.1%)	8 (0.6%)	<0.001
AIDS	112 (0.2%)	2 (0.2%)	0.878**
Lymphoma	301 (0.6%)	13 (1.0%)	0.047
Metastatic cancer	756 (1.4%)	58 (4.3%)	<0.001
Solid tumor w/out metastasis	1,539 (2.8%)	82 (6.0%)	<0.001
Rheumatoid arthritis	169 (0.3%)	4 (0.3%)	1.000**
Weight loss	1,140 (2.1%)	139 (10.2%)	<0.001
Deficiency anemias	462 (0.8%)	9 (0.7%)	0.475
Psychoses	2,013 (3.7%)	39 (2.9%)	0.123
Depression	5,021 (9.1%)	92 (6.8%)	0.003

*For congestive heart failure, pulmonary circulation disease, peripheral vascular disease, hypertension, diabetes w/o chronic complications, diabetes w/ chronic complications, liver disease, peptic ulcer disease bleeding, coagulopthy, obesity, fluid and electrolyte disorders, chronic blood loss anemia, alcohol abuse, and drug abuse, no individual was found.

**Yates correction is applied for those violated the assumption that 5 or more in all cells of a 2-by-2 table.

### Predictors of in-hospital mortality

After excluding LOS outliers, the bivariate logistic regressions were conducted between mortality and the independent variables as shown in Tables 2 and 3. From the results of the univariate analyses, the mortality risk of patients of COPD increased by 4% for every increase in age by 1 year and female patients were 19% less likely to die compared to male patients. The highest risk of mortality was in the patients who were Asian (odds ratio [OR] = 1.65; confidence interval [CI] = 1.02-2.65), had other insurance (OR = 3.31; CI = 2.63-4.16), were admitted with elective admission (OR = 1.95; CI = 1.67-2.27), had extreme severity of illness (OR = 38.26; CI = 29.61-49.43), had extreme risk of mortality (OR = 88.56; CI = 67.97-115.39), and were assigned with DCI score of 4 and above (OR = 2.98; CI = 2.50-3.55). Elixhauser comorbidities including valvular disease (OR = 1.50; CI = 1.07-2.10), other chronic lung disease (OR = 7.62; CI = 4.53-12.80), renal failure (OR = 6.39; CI = 3.03-13.46), metastatic cancer (OR = 3.24; CI = 2.45-4.27), solid tumor without metastasis (OR = 2.10; CI = 1.66-2.67), and weight loss (OR = 5.26; CI = 4.34-6.37) were more likely to happen to patients who died than who survived; however, comorbid conditions such as hypothyroidism (OR = 0.82; CI = 0.61-0.98) and depression (OR = 0.68; CI = 0.54-0.85) were associated with the decreased risk of morality. Oral/parenteral corticosteroids (OR = 0.76; CI = 0.66-0.88) and antibiotics (OR = 0.65; CI = 0.56-0.75) had Odds Ratio less than 1.0, which indicated that they had a protective effect on mortality. However, β-blockers (OR = 0.99; CI = 0.88-1.10) were not found to be significantly related to morality.

**Table 2:** **Univariate analysis of in-hospital mortality (N = 55,967)**

Characteristics	Unadjusted OR	95% Confidence interval	P-value
Age (increase by 1 year)	1.04	1.04-1.05	0.003
Gender			
Male	1.0	-	<0.001
Female	0.81	0.73-0.91	
Race/ethnicity			
Caucasian	1.0	-	<0.001
African American	0.76	0.62-0.94	
Hispanic	0.40	0.23-0.69	
American Indian	0.27	0.07-1.09	
Asian	1.65	1.02-2.65	
Other race	1.04	0.88-1.22	
Insurance type			
Medicare	1.0	-	<0.001
Medicaid	0.45	0.34-0.61	
Managed care	0.58	0.46-0.73	
Commercial	1.44	1.13-1.84	
Other	3.31	2.63-4.16	
Uninsured	0.46	0.30-0.72	
Hospital			
Urban	1.0	-	0.187
Rural	0.90	0.77-1.05	
Non-teaching	1.0	-	0.621
Teaching	1.03	0.92-1.16	
Number of beds	1.0	1.00-1.00	0.503
Admission type			
Emergency	1.0	-	<0.001
Urgent	1.12	0.97-1.30	
Elective	1.95	1.67-2.27	
Trauma	1.29	0.57-2.91	
ICU admission	8.82	7.87-9.88	<0.001
DRG severity of illness			
Minor	1.0	-	<0.001
Moderate	1.37	1.04-1.80	
Major	3.91	3.02-5.06	
Extreme	38.26	29.61-49.43	
DRG risk of mortality			
Minor	1.0	-	<0.001
Moderate	4.37	3.33-5.73	
Major	14.44	11.10-18.79	
Extreme	88.56	67.97-115.39	
LOS (increase by 1 day)	1.11	1.10-1.12	<0.001
Number of discharges	1.27	1.19-1.35	<0.001
Oral/parenteral corticosteroids	0.76	0.66-0.88	0.002
Antibiotics	0.65	0.56-0.75	<0.001
β-blockers	0.99	0.88-1.10	0.827

**Table 3:** **Univariate analysis of in-hospital mortality (N = 55,967)**

Characteristics	Unadjusted OR	95% Confidence interval	P – value
DCI score			
0	1.0	-	<0.001
1	1.57	1.35-1.82	
2	1.83	1.55-2.16	
3	2.21	1.82-2.69	
4+	2.98	2.50-3.55	
Elixhauser comorbidity			
No comorbidity	1.0	-	-
Valvular disease	1.50	1.07-2.10	0.026
Paralysis	0.71	0.18-2.86	0.607
Neurological disorders	1.12	0.78-1.61	0.537
Other chronic lung disease	7.62	4.53-12.80	<0.001
Hypothyroidism	0.82	0.68-0.98	0.021
Renal failure	6.39	3.03-13.46	<0.001
AIDS	0.38	0.05-2.69	0.247
Lymphoma	1.71	0.96-3.05	0.095
Metastatic cancer	3.24	2.45-4.27	<0.001
Solid tumor w/out metastasis	2.10	1.66-2.67	<0.001
Rheumatoid arthritis	1.00	0.37-2.69	1.000
Weight loss	5.26	4.34-6.37	<0.001
Deficiency anemias	0.65	0.31-1.37	0.218
Psychoses	0.73	0.52-1.03	0.058
Depression	0.68	0.54-0.85	<0.001

*For congestive heart failure, pulmonary circulation disease, peripheral vascular disease, hypertension, diabetes w/o chronic complications, diabetes w/ chronic complications, liver disease, peptic ulcer disease bleeding, coagulopthy, obesity, fluid and electrolyte disorders, chronic blood loss anemia, alcohol abuse, and drug abuse, no individual was found.

Since the variables APR-DRG severity of illness, APR-DRG risk of mortality, and DCI were highly correlated, we removed the variable APR-DRG severity of illness and APR-DRG risk of mortality from the multiple logistic regression models. The Spearman correlation coefficients are displayed in Table 4. All other independent variables significantly predictive of mortality (p ≤ 0.05) in the bivariate logistic regressions were retained for multivariate analyses.

**Table 4:** **Spearman correlation test for APR-DRG severity of illness, APR-DRG risk of mortality, and DCI**

		APR-DRG* severity of illness	APR-DRG* risk of mortality	DCI
APR-DRG* severity of illness	Correlation Coefficient	1.000	0.751	0.439
	P-value	-	<0.001	<0.001
APR-DRG* risk of mortality	Correlation Coefficient	0.751	1.000	0.499
	P-value	<0.001	-	<0.001

*APR-DRG: All Patient Refined Diagnosis Related Groups.

The multivariate analyses between all cause, in-hospital mortality for COPD patients and the independent variables are shown in Table 5. Two models were created for analysis, because DCI and Elixhauser comorbidities, which are two algorithms for assessing comorbidities, were analyzed in separate models. The goodness of fit Hosmer-Lemeshow tests indicated that both models fit reasonably well (p = 0.14 in the Elixhauser model; p = 0.23 in the DCI model). In collinearity tests, we did not find significant collinearity among the independent variables in each model. In both models, the mortality risk of patients of COPD increased by 6% for every 1 year increase in age. The highest risk of mortality was among patients who had: Asian race, insurance in the category defined as “other”, an elective admission, an ICU admission, and DCI score of 4 and above. The Elixhauser comorbidities of renal failure, metastatic cancer, solid tumor without metastasis, and weight loss were predictive of mortality; while comorbid conditions of hypothyroidism and depression were associated with the decreased risk of morality. Oral/parenteral corticosteroids, β-blockers and antibiotics had odds ratio less than 1.0, which indicated that they were associated with lower mortality. Gender was significantly associated with mortality in the DCI model, but was not significant in the Elixhauser model. Valvular disease and other chronic lung diseases that were significant in bivariate analysis were not significant predictors in the multivariable model. However, the use of β-blockers that was not significant in the bivariate analysis, was associated with lower mortality risk after adjusting for patient age, gender, race/ethnicity, insurance type, and comorbidities.

**Table 5:** **Multivariate analyses of all cause, in-hospital mortality of COPD patients (N = 55,967)**

Characteristic	Elixhauser model*				DCI model**			
	Adjusted OR	95% Confidence interval	Trend p-value	R-square	Adjusted OR	95% Confidence interval	Trend-p-value	R-square
Age (increase by 1 year)	1.06	1.05-1.07	<0.001	0.0060	1.06	1.05-1.07	<0.001	0.0060
Gender								
Male	-	-	-		1.0	-	0.029	<0.0001
Female	-	-			0.88	0.78-0.98		
Race/ethnicity								
Caucasian	1.0	-	0.005	0.0002	1.0	-	0.003	0.0004
African American	0.79	0.63-0.98			0.78	0.62-0.97		
Hispanic	0.43	0.25-0.75			0.41	0.23-0.71		
American Indian	0.35	0.09-1.43			0.34	0.08-1.38		
Asian	1.29	0.77-2.16			1.27	0.76-2.12		
Other race	0.91	0.77-1.08			0.91	0.77-1.08		
Insurance type								
Medicare	1.0	-	<0.001	0.0031	1.0	-	<0.001	0.0031
Medicaid	1.12	0.81-1.55			1.11	0.80-1.54		
Managed care	1.13	0.88-1.44			1.15	0.90-1.47		
Commercial	2.46	1.89-3.20			2.48	1.91-3.23		
Other	5.82	4.53-7.49			5.99	4.66-7.70		
Uninsured	1.41	0.87-2.24			1.56	0.99-2.48		
Admission type								
Emergency	1.0	-	<0.001	0.0009	1.0	-	<0.001	0.0010
Urgent	1.13	0.96-1.32			1.15	0.99-1.35		
Elective	1.86	1.57-2.19			1.95	1.65-2.30		
Trauma	1.70	0.74-3.92			1.76	0.76-4.05		
ICU admission	8.08	7.09-9.20	<0.001	0.0204	7.95	6.99-9.05	<0.001	0.0204
LOS (increase by 1 day)	1.06	1.05-1.07	<0.001	0.0027	1.06	1.05-1.07	<0.001	0.0027
Number of discharges	1.33	1.24-1.42	<0.001	0.0009	1.32	1.23-1.41	<0.001	0.0010
Therapies								
β-blockers	0.76	0.67-0.86	0.011	0.0003	0.68	0.60-0.95	<0.001	0.0006
Corticosteroids	0.82	0.70-0.95	<0.001	0.0001	0.82	0.70-0.95	0.010	0.0001
Antibiotics	0.54	0.46-0.63	<0.001	0.0013	0.55	0.47-0.65	<0.001	0.0012
DCI score								
0	-	-	-		1.0	-	<0.001	0.0010
1	-	-			1.35	1.16-1.58		
2	-	-			1.46	1.22-1.74		
3	-	-			1.54	1.25-1.90		
4+	-	-			2.18	1.80-2.64		
Elixhauser comorbidity								
Hypothyroidism	0.73	0.61-0.88	0.001	0.0002	-	-	-	
Renal failure	5.12	2.29-11.43	<0.001	0.0003	-	-	-	
Metastatic cancer	3.07	2.28-4.13	<0.001	0.0009	-	-	-	
Solid tumor w/out metastasis	1.72	1.33-2.24	<0.001	0.0003	-	-	-	
Weight loss	2.62	2.10-3.26	<0.001	0.0013	-	-	-	
Depression	0.78	0.62-0.98	0.034	0.0001	-	-	-	

*ORs are adjusted for all variables in the table except for DCI scores. The p value for good of fitness Hosmer-Lemeshow test is 0.142, and R-squared value is 0.039.

**ORs are adjusted for all variables in the table except for Elixhauser comorbidities. The p value for good of fitness Hosmer–Lemeshow test is 0.23, and R-squared value is 0.037.

An interaction between insurance type and admission type was found in both models. After adjusting the interaction, we found that the estimates of other independent variables were similar for each model. To define the source of the interaction we ran the model by each admission type (emergency, urgent, elective, and trauma). Among patients with emergency or trauma admissions, insurance type was not an independent predictor of in-hospital mortality. Among patients with urgent admissions, patients in the “other” insurance category had the highest risk of morality (Elixhauser model: OR 16.14 95% CI 10.12-25.75; DCI model: OR 16.44, 95% CI 10.34-26.14), followed by patients with commercial insurance (Elixhauser model: OR 4.45 95% CI 2.61-7.58; DCI model: OR 4.61, 95% CI 2.72-7.82) and, then managed care (Elixhauser model: OR 1.95, 95% CI 1.10-3.43; DCI model: OR 1.88, 95% CI 1.06-3.32). Among patients with elective admissions, patients in the “other” insurance category had the highest risk of morality (Elixhauser model: OR 12.80, 95% CI 7.44-22.04; DCI model: OR 13.89, 95% CI 8.07-23.93), followed by patients with commercial insurance (Elixhauser model: OR 7.25, 95% CI 4.38-12.00; DCI model: OR 7.41, 95% CI 4.49-12.22), no insurance (Elixhauser model: OR 3.83, 95% CI 1.46-10.03; DCI model: OR 4.85, 95% CI 1.92-12.27), and managed care (Elixhauser model: OR 2.10, 95% CI 1.15-3.83; DCI model: OR 2.11, 95% CI 1.16-3.85).

In order to control familywise error rates caused by these multiple comparisons, we used a Bonferroni correction to get adjusted p-values for independent variables in each model. After Bonferroni corrections, gender, African American race, and corticosteroids were not significantly associated with mortality in the DCI model; and race/ethnicity, corticosteroids, and depression were not associated with mortality any longer in the Elixhauser model.

## Discussion

All-cause, in-hospital mortality in COPD patients was 2.4%; similar to the reported rate of 2.5% based on the data from the 1996 U.S. Nationwide Inpatient Sample (Patil et al. 2003). We identified predictors for all-cause in-hospital mortality in COPD patients from the 2007 Premier Perspective database. Before this study, we conducted a literature review of predictors for mortality among patients with COPD and found that previous articles studied predictors primarily based on physiologic data (Cheng et al. 2011). However, in this study we identified significant predictors that included patients’ socio-demographic and medical characteristics, as well as hospital-specific characteristics. Although patient laboratory test results and physiologic indicators are not included in the Premier database, our findings may help health care providers identify COPD patients who would be more likely to die when admitted to the hospital. In addition, the study has several other advantages including a large sample size, use of population-based data, and inclusion of pharmacotherapy during the hospital stay.

In this study, we explored the ability of both the DCI and Elixhauser comorbidity measures to predict mortality in COPD patients. Compared to the DCI, the Elixhauser comorbidity measure does not assign a weight for each comorbid condition or give a summary score for comorbidity severity, but allows identification of specific comorbid conditions associated with in-hospital mortality. Although it was not our purpose to compare the two algorithms for comorbidities to predict health outcomes and both Elixhauser comorbidity measure and the DCI improved the predictive ability of the models we note the following differences. The DCI had a dose–response association with increased risk of in-hospital mortality in this study. The Elixhauser comorbidity measure identified specific comorbid conditions associated with significantly increased mortality risk; including renal failure, metastatic cancer, solid tumor without metastasis, and weight loss. COPD patients with hypothyroidism had significantly lower mortality risk, and this finding was consistent with some studies that have confirmed that subclinical hypothyroidism was associated with reduced all-cause mortality (Sathyapalan et al. 2010; Razvi et al. 2008; Gussekloo et al. 2004). It was reported previously that elderly individuals with higher thyrotropin-stimulating hormone had a longer life expectancy than those with normal levels (Gussekloo et al. 2004). Our study sample had an average age of 69.7 years old, so it was not out of expectation to see hypothyroidism had a protective effect on all-cause mortality of COPD patients. Although the recently published systematic review identified diabetes mellitus and congestive heart disease as the predictors of increased mortality in hospitalized COPD patients (Singanayagam et al. 2013), neither was statistically significant in our study.

Similar to the previous studies, gender was a controversial factor associated with mortality risk in the study. In the Elixhauser model, gender was not a significant factor to predict mortality, but in the DCI model, males had significantly higher in-hospital mortality risk than females. This finding was also consistent with COPD mortality odds ratio of male (OR = 1.17, CI = 1.07-1.27) reported.(5) However, after making the Bonferroni correction, gender was no longer a predictor of in-hospital mortality in the DCI model.

While previous studies have rarely reported race as a predictor of mortality among COPD patients, our results showed race-related differences. African Americans and Hispanics had a significantly lower risk of death than Caucasians in the both models, but only Hispanics remained statistically significant after Bonferroni correction in the DCI model. A review of COPD in Hispanics had a similar finding; lower mortality rates for COPD among Hispanics compared to non-Hispanics at each time point in the study period (Brehm and Celedon 2008). A recently issued report also confirmed that compared to non-Hispanic Whites and Blacks, Hispanics had a much lower age-adjusted death in both male and female patients with COPD (American Lung Assocation 2013).

Health insurance types were found to be related to morality risk. Patients with commercial and other insurances (including workers compensation, direct employer contract, and other government payers) had a significantly higher risk for mortality than those with Medicare or Medicaid. The out-of-pocket expenditure usually may be greater under commercial insurance than under public insurance such as Medicare and Medicare (Ku and Broaddus 2008). Patients under private insurance might hesitate to consume healthcare services in the early stage of disease, therefore hospitalized patients under private insurance would be more likely have worse health outcomes than those under public insurance. This explanation could be related to the results of our sub-analyses by admission type. We found insurance was only associated with decreased risk of in-hospital mortality among patients with urgent or elective admissions who had less severe medical condition than patients with emergency admissions. Insurance coverage was predictive for the patients with urgent or elective admission but not for emergency admissions. Elective admission was found to be associated with a higher risk of mortality compared to emergency admission. This was different from what was found in the previous studies and our hypothesis that patients admitted from an emergency department might have higher acuity of disease, placing them at a higher risk of mortality (Patil et al. 2003). Although the reason behind the phenomenon is unclear, there would be several possible explanations. One is that physicians in emergency room have experience in correctly diagnosing disease and appropriately treating patients. Emergency rooms are usually well equipped with medical professionals and health care resources to resuscitate patients with COPD exacerbations. As for elective admission, patients have to wait several days for obtaining authorization before they receive in-hospital care, so that COPD severity would get worse when they are admitted. So, another explanation is that patients who had elective admission might have more severe COPD than patients who had emergency admission due to the delayed care. The third is potentially related to coding and reporting errors. Since more than 600 hospitals contribute to the database, there may be inconsistent coding between hospitals .(Premier research service 2010) The study shows that oral/parenteral corticosteroids, antibiotics, and β-blockers were associated with decreased mortality risk in the both models, but after the Bonferroni correction corticosteroids were no longer associated with mortality risk. Although patients who were treated with these therapies may have a more severe disease, these treatments appeared to lower mortality. Several studies have verified that using antibiotics could reduce treatment failure and mortality in patients with acute exacerbations of COPD and patients with moderate or severe COPD (Evensen 2010; Stefan et al. 2013; Roede et al. 2009). Other studies have also found β-blockers have benefits for reducing mortality of COPD patients by reducing cardiovascular risk (Short et al. 2011; Tashkin 2012; Rutten et al. 2010). Since use of these therapies has shown promising beneficial effects on survival in COPD patients, prescribing these therapies should be studied in prospective research and possibly incorporated into clinical practice to manage COPD patients at high risk of mortality.

Several potential limitations were considered. First, the Premier database and its participants represented only the nationwide group of hospitals with the willingness and infrastructure to submit and track the data needed for decisions about Medicare payments (Premier research service 2010) Thus our study results may have limited generalizability. Second, we included only the patient’s primary discharge ICD-9-CM diagnosis if related to COPD. There could be misclassification of COPD with another respiratory illness such as asthma or cardiac disease, such as congestive heart failure. In addition, no spirometric data or inhaler use information was available in database to support the diagnosis. However, only including patients who were at least 40 years old could control misclassification of disease status, since misclassification would be more likely to occur among young patients. Third, some important predictors of mortality identified in the literature reviews such as FEV1, dyspnea, arterial blood gas measure, quality of life, body mass index, exercise tolerance, and inflammation were not available in the Premier database. However, we included other important information regarding comorbity such as the DCI and Elixhauser comorbidities to predict mortality. We also included hospital, admission information and health insurance status, which were rarely evaluated in previously published studies. Fourth, APR-DRG severity of illness and APR-DRG risk of mortality were found to be strong predictors for mortality in the bivariate analyses, but we did not include them in the final models in order to obtain comparative results with previous studies. These were rarely used in published articles however DCI and comorbid conditions reflect the severity of disease. Fifth, the data were analyzed on the last discharge of patients in year 2007. Since this was a cross-sectional study rather than a longitudinal study, information from previous years was not available, for instance, number of hospitalizations in previous years. Sixth, duration and dosage of each therapy were not available. Finally, many unobservable factors might bias the study results, such as smoking status, socioeconomic status, and severity of COPD exacerbation.

## Conclusion

In a study based on the nationwide discharge database, we found that older age, commercial and other insurance, elective admission, ICU admission, extended LOS, increased number of discharges, high DCI score, and Elixhauser comorbidities of renal failure, metastatic cancer, solid tumor without metastasis and weight loss, were associated with increased risk of all-cause in-hospital mortality of patients with COPD. However, antibiotics and β-blockers were associated with decreased mortality risk. We suggest that health care providers should consider these factors when assigning health resources to patients with COPD.

## Endnote

^a^APR-DRG is a clinical model developed by 3 M Health Information System Inc. that adds two sets of subclasses to the basic DRG structure about severity of illness and risk of mortality. DRG is a statistical system of classifying an inpatient stay into groups for the purposes of payment. DRG is used to reflect the average amount of resources needed to serve a specific patient. For each discharge, a patient is assigned to one DRG.

## Electronic supplementary material

Additional file 1: **Calculated mean and standard deviation of LOS for each DRG code.** (DOCX 15 KB)

## Abbreviations

COPD: Chronic obstructive pulmonary disease
FEV1: Forced expiratory volume in one second
BMI: Body mass index
LOS: Length of stay
DCI: Deyo-adapted Charlson comorbidity index
DRG: Diagnosis related groups
APR-DRG: All patient refined diagnosis related groups
ICD-9-CM: International Classification of Disease, Ninth Revision, Clinical Modification
IQR: Interquartile range.
