# Supplementary material for: Predictors for mortality in hospitalized patients with chronic obstructive pulmonary disease
Source: Springerplus. 2014 Jul 15;3:359. doi: 10.1186/2193-1801-3-359 (PMC4108717; doi:10.1186/2193-1801-3-359)
Supplement: Supplementary file 1 — Additional file 1: Calculated mean and standard deviation of LOS for each DRG code. (DOCX 15 KB) [file 40064_2014_1054_MOESM1_ESM.docx]

**Additional file 1.** Calculated mean and standard deviation of LOS for each DRG code

| DRG code | DRG Title | N | LOS mean(± s.d.) | Mean+4s.d. |
| --- | --- | --- | --- | --- |
| 75 | Major chest procedures | 202 | 9.45(± 7.34) | 38.79 |
| 76 | Other respiratory system operating room procedures with complication, comorbidities | 419 | 10.62(± 8.50) | 44.00 |
| 77 | Other respiratory system operating room procedures without complications, comorbidities | 23 | 4.91(± 2.81) | 16.15 |
| 88 | Chronic obstructive pulmonary disease | 53,878 | 4.48(± 3.40) | 18.08 |
| 96 | Bronchitis & asthma age >17 with complications, comorbidities | 3 | 3.33(± 1.53) | 9.44 |
| 97* | Bronchitis & asthma age >17 without complications, comorbidities | 1 | - | - |
| 468 | Extensive operating room procedure unrelated to principal diagnosis | 445 | 12.58(± 9.43) | 50.30 |
| 476 | Prostatic o.r. procedure unrelated to principal diagnosis | 15 | 15.07(± 8.81) | 50.31 |
| 477 | Non-extensive operating room procedure unrelated to principal diagnosis | 97 | 10.14(± 11.27) | 55.24 |
| 495 | Lung transplant | 27 | 16.00(± 9.46) | 53.85 |
| 541 | Ecmo or tracheostomy with mechanical ventilation 96+ hours or principal diagnoses except face, mouth and neck diagnoses with major operating room procedure | 30 | 37.73(± 23.07) | 130.01 |
| 542 | Tracheostomy with mechanical ventilation 96+ hours or principal diagnosis except face, mouth and neck diagnoses without major operating room procedure | 107 | 28.47(± 20.28) | 109.58 |
| 565 | Respiratory system diagnosis with ventilator support 96+ hours | 371 | 16.55(± 10.36) | 57.92 |
| 566 | Respiratory system diagnosis with ventilator support <96 hours | 682 | 8.84(± 7.21) | 37.683 |

* For DRG code 97, there was only 1 observation in the whole study.
